# Supplementary material for: Cu-Doped-ZnO Nanocrystals Induce Hepatocyte Autophagy by Oxidative Stress Pathway
Source: Nanomaterials (Basel). 2021 Aug 17;11(8):2081. doi: 10.3390/nano11082081 (PMC8399041; doi:10.3390/nano11082081)
Supplement: Supplementary file 1 [file nanomaterials-11-02081-s001.zip › nanomaterials-1325765-supplementary.pdf]

Supplementary Materials

Cu-doped-ZnO Nanocrystals Induce Hepatocyte Autophagy by Oxidative Stress Pathway

Qianyu Bai [1,†], Yeru Wang [2,†], Luoyan Duan [1,†], Xiaomu Xu [1], Yusheng Hu [1], Yue Yang [1], Lei Zhang [2], Zhaoping Liu [2], Huihui Bao [2,*] and Tianlong Liu [1,*]

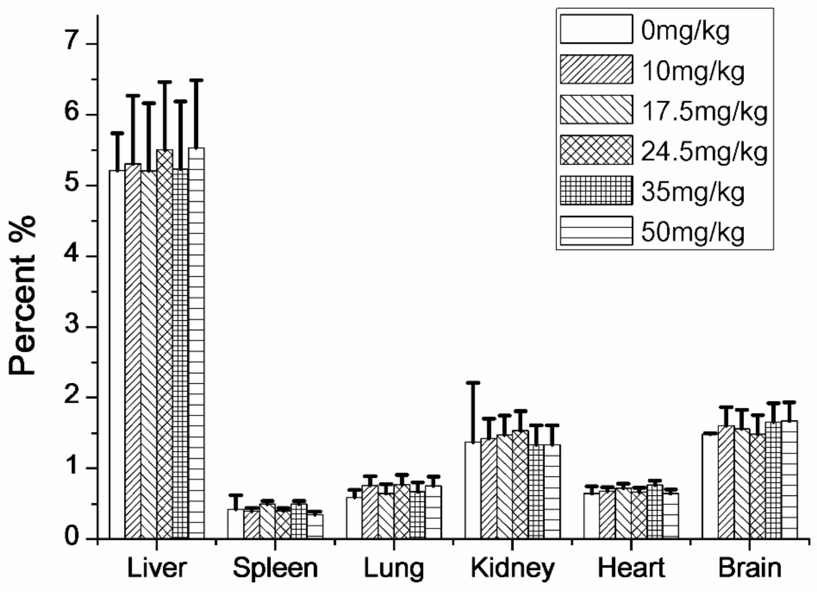

Figure S1. Coefficients indexes of major organs of mice received CZON.

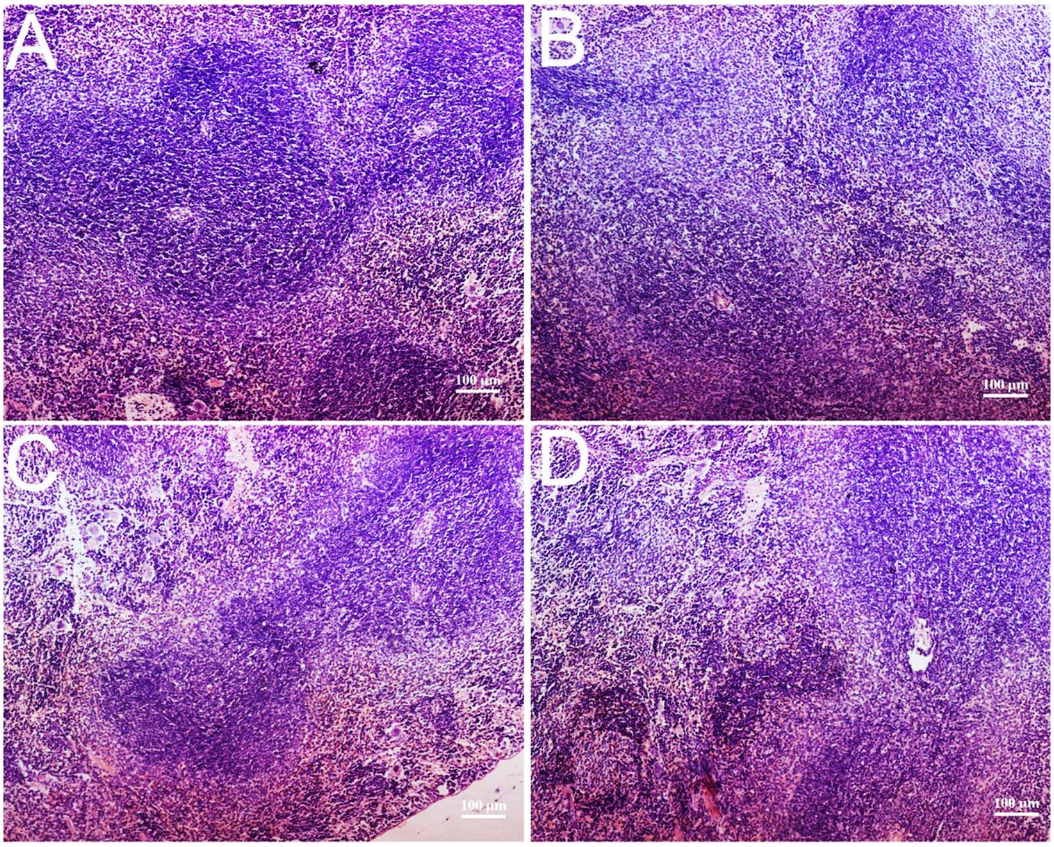

Figure S2. Histopathological sections of ICR mice; **(A)** the group of 200mg/kg; **(B)** the group of 100mg/kg; **(C)** the group of 50mg/kg; **(D)** the group of 25mg/kg. No obvious changes were observed of spleen in mice received CZON at all dosages compared to the control mice.

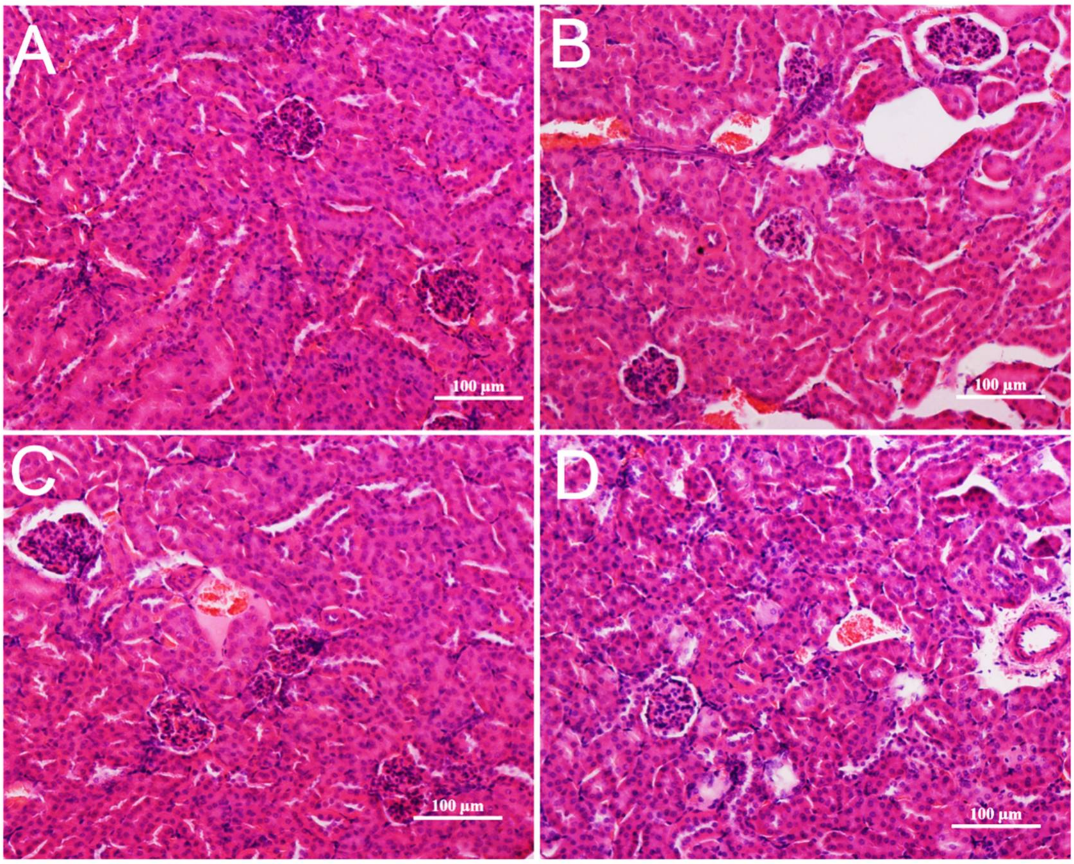

Figure S3. Histopathological sections of ICR mice; **(A)** the group of 200mg/kg; **(B)** the group of 100mg/kg; **(C)** the group of 50mg/kg; **(D)** the group of 25mg/kg. No obvious changes were observed of kidney in mice received CZON at all dosages compared to the control mice.

Table S1. probing study of CZON in mice after single injection.

Dosage mg/kg	Total No.	Death
200	10	5
100	10	3
50	10	0
25	10	0

Table S2. Body weight of mice received CZON at different dosages.

Groups	0 days	1 days	2 days	3 days	7 days	10 days	15 days
Con	24.44 ± 0.98	24.96 ± 1.32	24.6 ± 0.88	24.74 ± 0.56	24.84 ± 0.75	26.35 ± 0.54	28.32 ± 1.32
10 mg/kg	23.52 ± 1.21	23.7 ± 0.87	23.8 ± 0.67	24.52 ± 0.78	24.48 ± 0.56	25.85 ± 0.76	26.88 ± 0.87
17.5 mg/kg	23.98 ± 1.12	24.08 ± 0.76	24.24 ± 0.76	24.5 ± 0.68	24.4 ± 0.65	25.94 ± 0.85	26.54 ± 0.88
24.5 mg/kg	23.6 ± 0.89	23.62 ± 0.72	24.12 ± 0.87	24.56 ± 0.86	25.12 ± 0.93	27.35 ± 0.83	28.24 ± 1.04
35 mg/kg	23.56 ± 0.88	22.7 ± 0.85	23 ± 0.78	23.72 ± 0.97	25.88 ± 0.76	25.65 ± 0.87	26.96 ± 0.98
50 mg/kg	24.08 ± 0.87	21.18 ± 0.67	23.48 ± 0.89	24.36 ± 0.58	25.32 ± 0.55	26.35 ± 0.97	26.78 ± 0.82
